# Syndecan‐3 enhances anabolic bone formation through WNT signaling

**DOI:** 10.1096/fj.202002024R

**Published:** 2021-03-26

**Authors:** Francesca Manuela Johnson de Sousa Brito, Andrew Butcher, Addolorata Pisconti, Blandine Poulet, Amanda Prior, Gemma Charlesworth, Catherine Sperinck, Michele Scotto di Mase, Ke Liu, George Bou‐Gharios, Robert Jurgen van 't Hof, Anna Daroszewska

**Affiliations:** ^1^ Department of Musculoskeletal and Ageing Science (formerly Department of Musculoskeletal Biology) Institute of Life Course and Medical Sciences (formerly Institute of Ageing and Chronic Disease) University of Liverpool Liverpool UK; ^2^ Department of Biochemistry IIB University of Liverpool Liverpool UK; ^3^ Department of Biochemistry and Cell Biology Stony Brook University Stony Brook NY USA; ^4^ Department of Clinical Biochemistry and Metabolic Medicine Liverpool University Hospitals NHS Foundation Trust Liverpool UK; ^5^ Department of Rheumatology Liverpool University Hospitals NHS Foundation Trust Liverpool UK; ^6^ Present address: International Centre for Life Newcastle University Newcastle UK; ^7^ Present address: ZeClinics Barcelona Spain

**Keywords:** bone, frizzled, osteoblast, Syndecan‐3, WNT

## Abstract

Osteoporosis is the most common age‐related metabolic bone disorder, which is characterized by low bone mass and deterioration in bone architecture, with a propensity to fragility fractures. The best treatment for osteoporosis relies on stimulation of osteoblasts to form new bone and restore bone structure, however, anabolic therapeutics are few and their use is time restricted. Here, we report that Syndecan‐3 increases new bone formation through enhancement of WNT signaling in osteoblasts. Young adult *Sdc3^−/−^* mice have low bone volume, reduced bone formation, increased bone marrow adipose tissue, increased bone fragility, and a blunted anabolic bone formation response to mechanical loading. This premature osteoporosis‐like phenotype of *Sdc3^−/−^* mice is due to delayed osteoblast maturation and impaired osteoblast function, with contributing increased osteoclast‐mediated bone resorption. Indeed, overexpressing *Sdc3* in osteoblasts using the *Col1a1* promoter rescues the low bone volume phenotype of the *Sdc3^−/−^* mice, and also increases bone volume in WT mice. Mechanistically, SDC3 enhances canonical WNT signaling in osteoblasts through stabilization of Frizzled 1, making SDC3 an attractive target for novel bone anabolic drug development.

**Abbreviations**
BC: bone chips
BFR/BS: bone formation rate per bone surface
BMAT: bone marrow adipose tissue
BMP: bone morphogenetic protein
BMSCs: bone marrow mesenchymal stromal cells
BV/TV: bone volume per tissue volume
C.Th: cortical thickness
E.Pm: endosteal perimeter
FGF: fibroblast growth factor
Fzd/FZD: frizzled
HB‐GAM: heparin‐binding growth molecule
HSPG: Heparan Sulfate Proteoglycan
LGRs: leucine‐rich repeat containing G protein‐coupled receptors
LRP: lipoprotein receptor‐related protein
MAR: mineral apposition rate
MCSF: macrophage colony stimulating factor
MMI: polar mean moment of inertia
MS/BS: mineralizing surface per bone surface
N.Oc/BS: number of osteoclasts per bone surface
N.Oc/BV: number of osteoclasts per bone volume
N.Oc/TV: number of osteoclasts per tissue volume
Oc.S/BS: osteoclast surface per bone surface
OC: osteoclast
P.Pm: periosteal perimeter
RANK: receptor activator of NFκB
RANKL: RANK ligand
RSPOs: R‐spondins
Sdc/SDC: Syndecan
SMI: structure model index
Tb.N: trabecular number
Tb.Pf: trabecular pattern factor
Tb.Sp: trabecular separation
Tb.Th: trabecular thickness
TRAcP: tartrate resistant acid phosphatase
WT: wild type

## 1 INTRODUCTION

Osteoporosis is the most common age‐related metabolic bone disorder affecting millions of people worldwide and predicted to rise as the population ages.^1^ Osteoporosis is characterized by low bone mass and impaired bone micro‐architecture, which predispose to fragility fractures, approximately 9 million annually.^2^ Only a small number of anabolic treatments is available, use of which is time‐limited and reserved for severe cases.^3^ As osteogenic therapeutics are superior to anti‐resorptives, due to their ability to restore bone mass and architecture, there is a pressing need to gain a better understanding of anabolic pathways leading to bone formation, so that they can inform novel drug development.

One of the most important signaling pathways inducing osteoblastogenesis and bone formation is WNT‐dependent.^4^ Mechanical loading of bone induces osteogenesis through the activation of the canonical (β‐catenin‐dependent) WNT‐signaling, subsequent to repression of sclerostin, a WNT inhibitor, expressed by mechanosensing osteocytes.^5^

The canonical WNT‐signaling pathway is activated by WNT ligands, which bind to the receptor complex comprised of frizzled (FZD) and the low density lipoprotein receptor‐related proteins 5/6 (LRP5/6) to indirectly inhibit phosphorylation of β‐catenin, allow for its translocation into the nucleus and induction of β‐catenin‐response genes including *AXIN2*.^4^ WNT signaling intensity depends on the availability of FZD receptors, the level of which is regulated by ubiquitin‐mediated degradation involving E3 ubiquitin ligases (ZNRF3 or RNF43), dependent on, or independent of leucine‐rich repeat containing G protein‐coupled receptors (LGRs).^6^, ^7^ In the presence of extracellular R‐spondins (RSPOs), which bind to LGRs, the RSPO‐LGR complex interacts with ZNRF3/RNF43 and prevents the latter from tagging FZD for degradation. Thus, the resulting high level of FZD allows for strong WNT‐induced signaling.^6^ Inactivating mutations of RSPO2, which interfere with RSPO2‐LGR or ‐RNF43 interaction, inhibit WNT signaling, and induce limb abnormalities in humans^7^ and mice.^8^ However, as deletion of LGRs does not recapitulate this phenotype, another, LGR‐independent, mechanism of regulation of FZD level has been proposed^7^ and evidence presented indicating that it may involve heparan sulfate proteoglycans (HSPGs), including syndecans (SDCs).^6^, ^9^ Interestingly, a gene expression study of human bone subject to mechanical loading revealed that some of the induced pathways are mediated by SDCs.^10^

SDCs are a family of four type 1 transmembrane HSPG receptors, which play a role in cell adhesion, signaling, and are co‐receptors for growth factors and their receptors. SDCs contain three domains: an N‐terminal ectodomain, a transmembrane‐, and a C‐terminal cytoplasmic domain. The ectodomain, which can be shed, mediates cell‐cell and cell‐matrix interaction through the attached glycosaminoglycan (GAG) chains including heparan sulfate (HS) in case of SDC2 and SDC4, and both HS and chondroitin sulfate (CS) in case of SDC1 and SDC3. The transmembrane domain allows for dimerization, whereas the cytoplasmic domain (composed of conserved regions C1 and C2 flanking a variable region [V]) interacts with kinases, the cytoskeleton and C1 mediates endocytosis.^11^, ^12^

GAGs, abundant in the extracellular matrix (ECM), are known to enhance bone regeneration and osteoblastogenesis by binding of the WNT inhibitor, sclerostin. However, with aging the amount of GAGs, and in particular CS decreases in human cortical mineralized bone matrix, which has a detrimental effect on bone toughness and likely contributes to the age‐related deterioration of bone quality.^13^ Previous research^14^ has shown that HS GAGs are important in osteoblast differentiation and activity, and SDCs with attached GAGs, can modulate WNT signaling,^15^, ^16^ however, SDC‐WNT cross talk in bone has not been clearly deciphered^4^, ^17^ and involvement of SDCs in bone physiology is poorly defined. SDC3 is expressed in the bone shafts of developing chick embryos^18^ and plays a complex role in regulating chondrocyte proliferation,^19^, ^20^ however, its exact role in skeletogenesis or bone homeostasis is not fully understood.

Here, we demonstrate that deletion of *Sdc3* leads to a premature low bone volume phenotype associated with increased bone fragility and increased bone marrow adipose tissue (BMAT) in young adult mice. The underlying mechanism involves impaired bone formation and attenuated anabolic bone response to mechanical loading, explained by impaired canonical WNT3a‐mediated signaling and likely due to increased degradation of FZD1 receptor in osteoblast‐lineage cells. Osteoblast‐specific overexpression of *Sdc3* not only restores the bone volume in *Sdc3^−/−^*, but also increases the bone volume in WT mice, which demonstrates an essential role of SDC3 in enhancing bone formation.

## 2 MATERIALS AND METHODS

### 2.1 Mice

Mice were housed in a pathogen‐free facility at the University of Liverpool, with free access to food and water, and in accordance with the Animals (Scientific Procedures) Act 1986 Amendment Regulations (SI 2012/3039) and the EU Directive 2010/63/EU, after the local ethical review and approval by Liverpool University's Animal Welfare and Ethical Review Body (AWERB). *Sdc3^−/−^* mice on C57Bl6 background^21^ were donated by Prof Heikki Rauvala, University of Helsinki, Finland. *Sdc3^−/−^* mice were generated from heterozygous and/or one generation homozygous breeding. Control wild‐type (WT) mice were littermates or closely related, age‐ and sex‐matched. To rescue the *Sdc3^−/−^* phenotype, *Col1a1‐Sdc3* mice on *Sdc3* null background were generated. The 2.3 kb *Col1a1* promoter vector driving osteoblast‐specific expression was kindly donated by Prof Gerard Karsenty, Columbia University, US.^22^ The vector was used to clone in the full *Sdc3* cDNA with *Myc* tag (1392 bp), IRES, and *mCherry* with a poly A (EX‐Mm05553‐M73, GeneCopoeia) using *NdeI* and *SacI* restriction enzymes. This final vector was linearized using *NruI* restriction to remove the bacterial selection sequences prior to pro nuclear injection of Day 1 *Sdc3^−/−^* embryos to generate the transgenic *Col1a1‐Sdc3* mice on *Sdc3* null background. Adult mice were genotyped for a series of transgenes including *Col1a1* promoter to *Sdc3* (F: TGGTATAAAAGGGGCCCAGG, R: CCGAATTCCTTCAAGCCTGC), within *Sdc3* (F: ATTAGCTGGCTCTCC, R: ACGGTAGTATGACTTGGGT), *Sdc3* to *mCherry* (F: GCGTCACGTACCAGAAACCT, R: ATGTTATCCTCCTCGCCCTTG) and within *mCherry* (F: CCCCGTAATGCAGAAGAAGA, R: TTGGTCACCTTCAGCTTGG). Three lines were obtained which showed appropriate integration of the vector, one of which was analyzed for this study. The *Col1a1‐Sdc3* mice were crossed with heterozygous *Sdc3^+/−^* mice to provide mice for bone phenotyping. Two of the WT and all the *Sdc3^−/−^* mice for this analysis were obtained from parallel heterozygous *Sdc3^+/−^* matings. The strategy outlining the generation of the *Col1a1‐Sdc3* mice is outlined in Figure S1.

### 2.2 In vivo mechanical loading

The right tibiae of 10‐week‐old male *Sdc3^−/−^* (n = 8) and WT (n = 7) mice were mechanically loaded to induce metaphyseal bone formation, as described.^23^ Briefly, mice were anesthetized using Isoflurane through the whole procedure. The right knee and ankle were placed in custom made cups in the ElectroForce® 3100 (TA Instruments, USA) and repetitive mechanical compression applied at a peak magnitude of 11N for 0.05 seconds. Baseline hold time of 2N was applied for 9.9 seconds to maintain the tibia in place in between peak load applications. Each loading episode consisted of 40 cycles and was applied six times over a period of 2 weeks. Mice received calcein (2 mg/mL) 150 µL intraperitoneal injections on days 8 and 12 (day 1 = first loading day). Mice were culled 3 days after the final loading episode and tibiae collected.

### 2.3 µCT analysis

The skin was removed, and hind limbs and spines were fixed overnight in 4% of formalin‐buffered saline and stored in 70% of ethanol. µCT analysis was performed using a Skyscan 1272 system (Bruker, Belgium). For assessment of bone morphometry, tibias, femurs, and the lumbar spine were dissected free of most soft tissue and scanned at a resolution of 4.5 µm (60 kV, 150 µA, rotation step size 0.3°, using a 0.5 mm aluminum filter). The reconstruction was performed using the Skyscan NRecon package. Trabecular parameters were measured using Skyscan CTAn software in a stack of 200 slices immediately proximal, or distal to the growth plate in femurs, or tibias, respectively, as described previously.^24^ Tibial and femoral cortical parameters were measured using Skyscan CTAn software in a stack of 100 slices immediately proximal to the tibiofibular junction and distal to the trochanter, respectively. Trabecular bone was separated from cortical bone using a CTAn macro, and the same macro then performed measurement of the cortical and trabecular parameters according to ASBMR guidelines.^25^ For tibial length quantification, the full length of mineralized tibial diaphysis at P2, and the distance between proximal and distal growth plate during skeletal maturation was measured on µCT longitudinal scans of 9 µm resolution using the Skyscan Data Viewer software.

For measurement of the whole bone volume of the tibial bone shaft of 2‐day‐old and 2‐week‐old mice, tibias were scanned at a resolution of 9 µm, and reconstructed as detailed above. Using a macro in CTAn, the bone was separated from soft tissues using a fixed threshold and bone volume measured between the proximal and distal growth plates.

Morphometry assessment was performed by an observer blinded to genotype.

### 2.4 Bone histomorphometry and histology

Bone samples were processed and stained for histology as previously described.^26^ Briefly, mice received intraperitoneal calcein injections (2 mg/mL, 150 µL) 5 days and 2 days before culling. The skin was removed, hind limbs were fixed for 24 hours in 4% of formalin, and stored in 70% of ethanol. The samples were embedded in methyl methacrylate (MMA) and 5 µm sections were cut using a tungsten steel knife on a Leica motorized rotary microtome. Sections were stained for TRAcP to visualize osteoclasts and counterstained with Aniline Blue. For analysis of calcein‐double labeling, sections were counterstained with Calcein Blue and histomorphometry performed as described previously.^26^ Sections were visualized on a Zeiss Axio Scan.Z1 using a ×10 lens (pixel size 0.45 µm) for TRAcP‐stained sections, and a ×20 lens and a pixel size of 0.23 µm for calcein labels. Histomorphometry was performed using the TrapHisto, OsteoidHisto, and CalceinHisto open‐source image analysis programs^26^ available at https://www.liverpool.ac.uk/ageing‐and‐chronic‐disease/bone‐hist/.

Adipocytes were quantified in sections stained with Goldner's Trichrome, imaged on the Zeiss Axio Scan.Z1 using a x10 lens, and quantified using an in‐house developed program (FatHisto) based on ImageJ. The presence of adipocytes in the MMA‐embedded sections was verified by immunostaining for perilipin on dewaxed paraffin‐embedded sections of tibiae from 3‐month‐old *Sdc3^−/−^* (n = 3) and WT (n = 3) mice. Sections were incubated in Unitrieve solution, blocked with goat serum, and adipocytes were detected using perilipin antibody (Abcam) and secondary goat anti‐rabbit Alexa Fluor 594 antibody (Invitrogen) at 1:500 and 1:200 dilution, respectively.

For analysis of the growth plate, mouse knees were decalcified in formical overnight, embedded in wax, and 5 µm sections cut on a Leica rotary microtome. Sections were stained for hematoxylin as well as Safranin O/fast Green, imaged using the Zeiss Axio Scan.Z1 using a ×10 lens, and growth plate width measured using Zeiss Zen software. Bone histomorphometry was performed by an observer blinded to genotype.

### 2.5 Three‐point bending test

Flexural strength of the femurs from 3‐month‐old *Sdc3^−/−^* and WT mice was assessed by dynamic three‐point bending test on freshly dissected, unfixed bones using the ZWICK‐mechanical testing machine (ZWICK, Switzerland), using a span of 8mm between the support points and a crosshead speed of 1 mm/min.

### 2.6 Osteoclast culture

Bone marrow was obtained from 2‐3‐month‐old mice and cultured with 100 ng/mL of M‐CSF (Prospec Bio) in αMEM (Gibco Invitrogen) supplemented with 10% of FCS for 3 days. Next, the adherent M‐CSF‐dependent macrophages were harvested and plated in 96‐well plates at 10^4^ cells/well in αMEM supplemented with 10% of FCS, 25 ng/mL of M‐CSF, and varying amounts of RANKL (R&D Systems), according to standard methods as previously described.^27^ After 5‐6 days of culture, the cells were fixed using 4% of paraformaldehyde in PBS, washed and stained for TRAcP. Numbers of osteoclasts were counted after TRAcP staining by an observer blinded to the genotype. Resorption assays were performed using the same method, but by plating the cells on dentine slices placed within 96‐well plates. After 6 days, the plates were fixed and stained for TRAcP, and number of osteoclasts per dentine slice counted. Next, the cell layer was polished off the dentine slices, and the entire slices imaged using an Olympus reflected light microscope (5× lens) fitted with a Zeiss Axiocam camera (isotropic pixel size 1.2 µm). Individual fields (8‐9 per slice) were stitched together using Microsoft ICE, and the pit area measured using ImageJ.

### 2.7 Osteoblast culture

Bone marrow was obtained from the long bones of 2‐3‐month‐old mice by removing the ends of the bones to expose the bone marrow cavity, followed by centrifugation at 300 g for 3 minutes. Bone marrow mesenchymal stromal cells (BMSCs) were cultured in DMEM (Gibco Invitrogen) supplemented with 10% of FCS and antibiotics (non‐osteogenic culture conditions). Osteoblasts were differentiated from BMSCs by culturing in osteogenic medium, DMEM supplemented with 10% of FCS, 1% of P/S, 2 mM of β‐glycerophosphate (Sigma‐Aldrich), and 50 µg/mL of 2‐Phospho‐L‐ascorbic acid trisodium salt (Sigma‐Aldrich). After removal of the bone marrow, the remaining femoral and tibial diaphysis were cut into small pieces followed by collagenase digestion for 30 minutes. The bone chips were washed in PBS and cultured in αMEM supplemented with 10% of FCS in 25 cm^2^ tissue culture flasks until semi confluent layers of osteoblasts (bone chip osteoblasts, BC) had grown out of the bone chips. The cultures were washed with PBS and the osteoblasts harvested using trypsin.

Calvarial osteoblasts were isolated from the calvaria of 2‐3‐day‐old pups by collagenase digestion as described in detail previously^28^ and cultured in αMEM supplemented with 10% of FCS and antibiotics.

For alkaline phosphatase assays, BC osteoblasts were plated at 15 × 10^3^ cells/well in 96‐well plates and cultured in 150 µL of αMEM with 10% of FCS for 24 hours. Alamar Blue (15 µL/well) was added to the culture and culture continued for 2 more hours. Cell viability was measured by analyzing the Alamar Blue signal using a fluorescence plate reader (BMG FLUO Star Optima, excitation 560 nm, emission 590 nm). Next, the cells were fixed for 10 minutes at 4°C using 4% of paraformaldehyde in PBS, washed and stored dry at −20°C. Alkaline phosphatase was measured using the conversion of para‐nitrophenyl‐phosphate by measuring absorption at 405 nm at 1 minute intervals for 30 minutes. Alkaline phosphatase measurements were corrected for cell number by dividing by the Alamar Blue signal.

For mineralization assays osteoblasts, either derived from bone chips or calvaria, were cultured for 26 days in mineralizing‐medium containing DMEM (Gibco Invitrogen) supplemented with 10% of FCS, 1% of P/S, 2 mM of β‐glycerophosphate (Sigma‐Aldrich), and 50 µg/mL of 2‐Phospho‐L‐ascorbic acid trisodium salt (Sigma‐Aldrich). Half medium changes were made every other day during induction period and mineralizing‐medium was made up fresh every time. Calcium deposition in the mineralization assay, at day 26, was measured using Alizarin Red staining. Osteoblasts were fixed in 70% of ice cold Ethanol for 1 hour on ice and washed in dH_2_O. Cells were then incubated in Alizarin Red S (Sigma‐Aldrich) solution for 20 minutes at room temperature on a rocking table, washed in dH_2_O overnight, and then, air dried. Quantification was performed by dissolving the alizarin Red in 10% (w/v) cetylpyridinium chloride in 10 mM of sodium phosphate, and measuring absorbance at 562 nm as previously described.^29^ For mineralization assays of BMSC, cells were grown to confluence in 12‐well plates in DMEM supplemented with 10% of FCS and antibiotics. Medium was changed to osteogenic medium and culture continued for 10 days with half medium changes every other day. Cultures were fixed and stained for Alizarin Red S as described above.

### 2.8 Adipo‐osteogenic culture of bone marrow stromal cells

BMSCs were isolated from mouse long bones as described above, plated at 2 × 10^6^ cells per cm^2^ in αMEM supplemented with 15% of FCS and grown until confluent (approximately 10 days) with twice weekly medium changes. After reaching confluence the medium was replaced with co‐differentiation adipogenic and osteogenic medium, αMEM supplemented with 10% of FCS, 2 mM of β‐glycerophosphate, 50 µg/mL of 2‐Phospho‐L‐ascorbic acid trisodium salt, and 100 nM of dexamethasone (Sigma‐Aldrich), adapted from Ghali et al.^30^ Culture was continued with 3‐weekly changes of medium for 12 more days, plates fixed, and adipocytes stained with Oil‐red‐O. After staining, the plates were imaged using a Nikon Diaphot inverted microscope fitted with a ToupTeK camera, using a 10× lens, and number of adipocytes per image field counted, counting at least three fields for every mouse cell donor.

### 2.9 Cell proliferation assay

Anti‐Ki67 (Abcam) was used to determine cell proliferation. BC‐derived osteoblasts from Sdc3*^−/−^* (n = 6) and WT (n = 7) mice were plated on glass cover slips (6 per mouse) in 24‐well plates, cultured for 48 hours, fixed in 100% of Methanol for 5 minutes, permeabilized with 0.1% of Triton‐X‐100 for 5 minutes, and then, blocked with 10% of FCS in PBS for 1 hour. The cells were then incubated with Anti‐Ki67 antibody at 1:250 dilution overnight at 4ºC. Fluorescent Alexa fluor‐488 conjugated antibody (Life Technologies) at 2 µg/mL was used for detection. Nuclei were stained with DAPI. The coverslips were imaged using a Zeiss Axio Scan.Z1 using a ×20 lens, and Ki‐67 positive and negative nuclei quantified using a macro in ImageJ.

### 2.10 qPCR

RNA was extracted from cell cultures using TRIzol. Reverse transcription was performed (1 µg RNA/sample) with three replicate RT reactions per sample, using Roche EvoScript according to the manufacturers protocol. quantitative polymerase chain reaction (qPCR) was performed on a Roche LightCycler 480 using Roche LightCycler Mastermix and validated Roche and Fisher Scientific TaqMan primer probe sets (Table S1). *Hmbs* was used as a house keeping gene, and expression was calculated as relative expression to this house keeping gene using the delta‐CT method. For experiments on the effects of WNT3a, cells were cultured as described until near‐confluence and after overnight serum starvation (where 10% FCS was replaced by 2% TCM), recombinant mouse WNT3a protein (Abcam) 50 ng/mL was added and cells cultured for 8 hours before lysis in TRIzol.

### 2.11 Western blotting

Cells were cultured as described above. After cell lysis using standard RIPA buffer, proteins were separated by sodium dodecyl sulfate‐polyacrylamide gel electrophoresis and electroblotted onto nitrocellulose membranes (Bio‐Rad). Membranes were blocked with blocking buffer (LI‐COR) and probed with primary antibodies to FZD1 (1 µg/mL, R&D), non‐phospho (active) β‐catenin, and GAPDH (1:1000, Cell Signaling). After washing with TBST, membranes were incubated with appropriate LI‐COR infrared‐labeled secondary antibodies, visualized using the Odyssey CLx imaging system (LI‐COR) and analyzed using Image Studio lite (LI‐COR, 5.2).

### 2.12 Statistical analysis

All experiments were performed with a minimum of three biological replicates, unless indicated otherwise. Statistical analysis was performed in SPSS using a (two‐tailed) Student's t test, unless indicated otherwise and a *P* < .05 was considered statistically significant.

## 3 RESULTS

### 3.1 Syndecan‐3 deletion results in a low bone volume phenotype in adult mice

**FIGURE 1:**
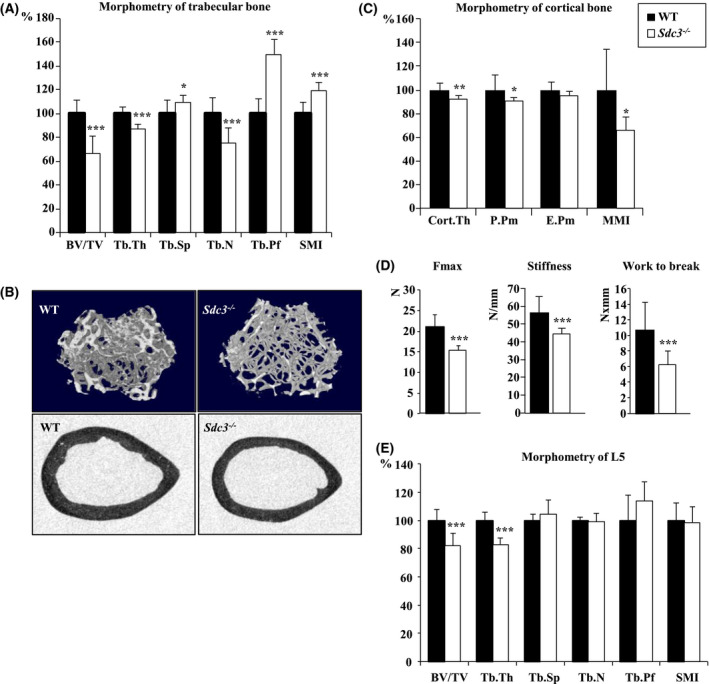
Syndecan‐3 enhances bone volume, architecture, and strength in adult mice. A, Morphometry of proximal tibiae of 3‐month‐old male *Sdc3^−/−^* (n = 13) and WT (n = 12) mice analyzed using µCT. BV/TV: bone volume, Tb.Th: trabecular thickness, Tb.Sp: trabecular separation, Tb.N: trabecular number, Tb.Pf: trabecular pattern factor, SMI: structure model index. B, Top panels: cross‐section images of 3D µCT reconstructions of proximal tibiae of *Sdc3^−/−^* vs WT mice representing the volume used for analysis of trabecular bone parameters. Bottom panels: representative cross‐sections at the mid shaft of the femur of a WT and *Sdc3* ^‐/‐^ mouse, showing decreased cortical thickness in the *Sdc3^−/−^* femur. All images µCT at 4.5 µm. C, Morphometry of tibial cortex of 3‐month‐old male *Sdc3^−/−^* (n = 10) and WT (n = 12) mice analyzed using µCT. C.Th: cortical thickness, E.Pm: endosteal perimeter, P.Pm: periosteal perimeter, MMI: polar mean moment of inertia. D, Three‐point bending test on femurs assessing the maximum force (Fmax), stiffness, and work to break in *Sdc3^−/−^* (n = 10) vs WT (n = 10) male mice. E, Morphometry of spine (L5) of 3‐month‐old male *Sdc3^−/−^* (n = 11) and WT (n = 11) mice. Data in a and c‐e are shown as mean ± SD. The data in panels A, C, and E are expressed as percentage of average WT. * *P < *.05, ** *P < *.01, *** *P < *.001

*Sdc3^−/−^* mice were viable, fertile, and showed no difference in weight vs WT. Morphometric µCT analysis of proximal tibiae of 3‐month‐old male *Sdc3^−/−^* mice revealed a decrease in bone volume (BV/TV), trabecular thickness (Tb.Th), and trabecular number (Tb.N) of 34% (*P < *.001), 13% (*P < *.001), and 24% (*P < *.001), respectively, and an increase in trabecular separation (Tb.Sp), trabecular pattern factor (Tb.Pf, indicating reduced connectivity), and structure model index (SMI, indicating a more rod‐like rather than plate‐like structure), of 10% (*P < *.05), 50% (*P < *.001), and 20% (*P < *.001), respectively, compared to WT mice (Figure 1A,B and Table S2A). Morphometry of distal femora of male mice, and tibias and femora from female mice showed similar results in keeping with a low trabecular bone volume phenotype in young adult *Sdc3^−/−^* mice (Figure 1B and Table S2A).

Cortical thickness (C.Th), periosteal perimeter (P.Pm), and polar mean moment of inertia (MMI) were decreased by 8% (*P < *.01), 9% (*P < *.05), and 34% (*P < *.05), respectively, in the tibiae of 3‐month‐old *Sdc3^−/−^* vs WT mice (Figure 1B,C and Table S2B). Morphometry of femoral cortex showed a decrease in C.Th, P.Pm, endosteal perimeter (E.Pm), and MMI of 11% (*P < *.001), 6% (*P < *.01), 12% (*P < *.001), and 21% (*P < *.01), respectively, in *Sdc3^−/−^* vs WT (Table S2B). As expected from the results of the µCT analysis, the three‐point bending test applied to femurs revealed a reduction in maximum force and work to fracture of 27% (*P < *.001) and 40% (*P < *.001), respectively, as well as a 20% reduction in stiffness (*P < *.001) in the *Sdc3^−/−^* vs WT mice (Figure 1D), indicating bone fragility in *Sdc3^−/−^* mice. Furthermore, morphometry at the lumbar spine also showed a decrease in BV/TV and Tb.Th in *Sdc3^−/−^* vs WT mice (Figure 1E). Thus, *Sdc3* deletion induces a low bone volume phenotype in both the trabecular and cortical compartments in adult mice, which is associated with bone fragility.

### 3.2 Syndecan‐3 differentially regulates prenatal and postnatal bone development

**FIGURE 2:**
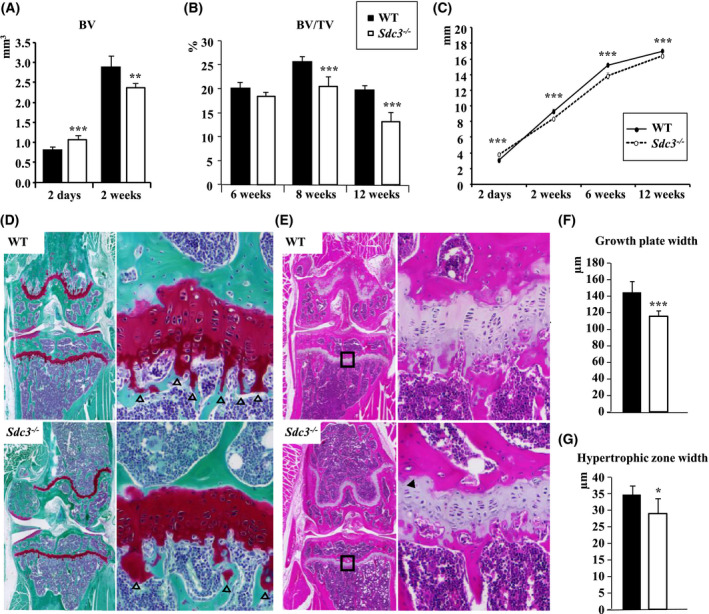
Syndecan‐3 differentially affects bone development in utero vs postnatally. A, Total bone volume of tibiae from 2‐day‐old pups (*Sdc3^−/−^* n = 9 vs WT n = 9) and 2‐week‐old male mice (*Sdc3^−/−^* n = 6 vs and WT n = 6) quantified using µCT. B, Trabecular bone volume per tissue volume (BV/TV) of the proximal tibia in males assessed by µCT during skeletal maturation at weeks: 6, 8 (at each time‐point *Sdc3^−/−^* n = 6 vs WT n = 6), and 12 (*Sdc3^−/−^* n = 13 vs WT n = 11). C, Length of tibial diaphysis quantified on µCT during skeletal maturation after birth at day 2 (*Sdc3^−/−^* n = 9 vs WT n = 9), week 2 (*Sdc3^−/−^* n = 6 vs WT n = 6), 6 (*Sdc3^−/−^* n = 5 vs WT n = 6), and 12 (*Sdc3^−/−^* n = 6 vs WT n = 5). All mice were male except pups at day 2 (undefined). D, Safranin‐O/fast green stain of knee sections of 3‐month‐old WT and *Sdc3^−/−^* mice. Regions of the growth plate delineated by the squares in the left panels have been magnified in the right panels. Primary spongiosa is indicated by arrowheads. GP—growth plate. E, H&E stain of knee sections of 3‐month‐old WT and *Sdc3^−/−^* mice. Regions of the growth plate delineated by the squares in the left panels have been magnified in the right panels. Hypertrophic region of the growth plate is indicated by dashed lines. Disorganized morphology of the growth plate in *Sdc3^−/−^* is indicated by an arrowhead. F, Quantification of the growth plate width in knee sections of 3‐month‐old male WT (n = 8) and *Sdc3^−/−^* (n = 4) mice. G, Quantification of the growth plate hypertrophic zone in knee sections of 3‐month‐old male WT (n = 8) and *Sdc3^−/−^* mice (n = 4). Data in a, b, c, f, and g are shown as mean ± SD. *P < .05; **P < .01; ***P < .001

To ascertain whether the low bone volume phenotype of adult *Sdc3^−/−^* mice was due to a developmental defect, we measured the total bone volume and length of tibiae just after birth and during skeletal maturation. Surprisingly, tibiae from 2‐day‐old *Sdc3^−/−^* pups had an approximately 40% higher bone volume vs WT (*P < *.001), however, by 2 weeks of age the *Sdc3^−/−^* mice tibia bone volume was approximately 20% lower than that of the WT (*P < *.01; Figure 2A). There was no difference in trabecular BV/TV between the genotypes at 6 weeks, however, as of 8 weeks of age the BV/TV was significantly lower in *Sdc3^−/−^* vs WT mice (*P < *.001; Figure 2B). This differential bone volume variation was mirrored by changes in the tibial longitudinal growth. Thus, at 2 days of age the tibiae of *Sdc3^−/−^* mice were 0.8 mm longer than those of WT (*P < *.001; Figure 2C), however, this difference was reversed already by 2 weeks, at which stage the *Sdc3^−/−^* tibiae were shorter by 0.9 mm vs WT (*P < *.001) and remained shorter throughout (Figure 2C). Taken together these data indicate that SDC3 has a differential effect on pre‐ and postnatal longitudinal bone development and growth. Moreover, there is no evidence that the low bone volume phenotype of 3‐month‐old *Sdc3^−/−^* mice could be due to a prenatal bone developmental defect.

As SDC3 is known to attenuate BMP2‐mediated limb chondrogenesis,^31^ we measured the growth plate width in the proximal tibia of 3‐month‐old mice, and found it thinner in *Sdc3^−/−^* vs WT mice, *P < *.001 (Figure 2D and F). Furthermore, the hypertrophic zone width was reduced, *P < *.05 (Figure 2E,G), and disorganized growth plate morphology was evident, with poorly stacked chondrocytes in irregular columns within the proliferating zone and a virtual absence of primary spongiosa (Figure 2D,E), which in part may explain the decreased long bone growth in young and adult *Sdc3^−/−^* mice, but not the low BV/TV in both trabecular and cortical compartment.

### 3.3 Low bone volume phenotype in adult *Sdc3^−/−^* mice is associated with impaired osteoclastogenesis, but increased osteoclast activity

**FIGURE 3:**
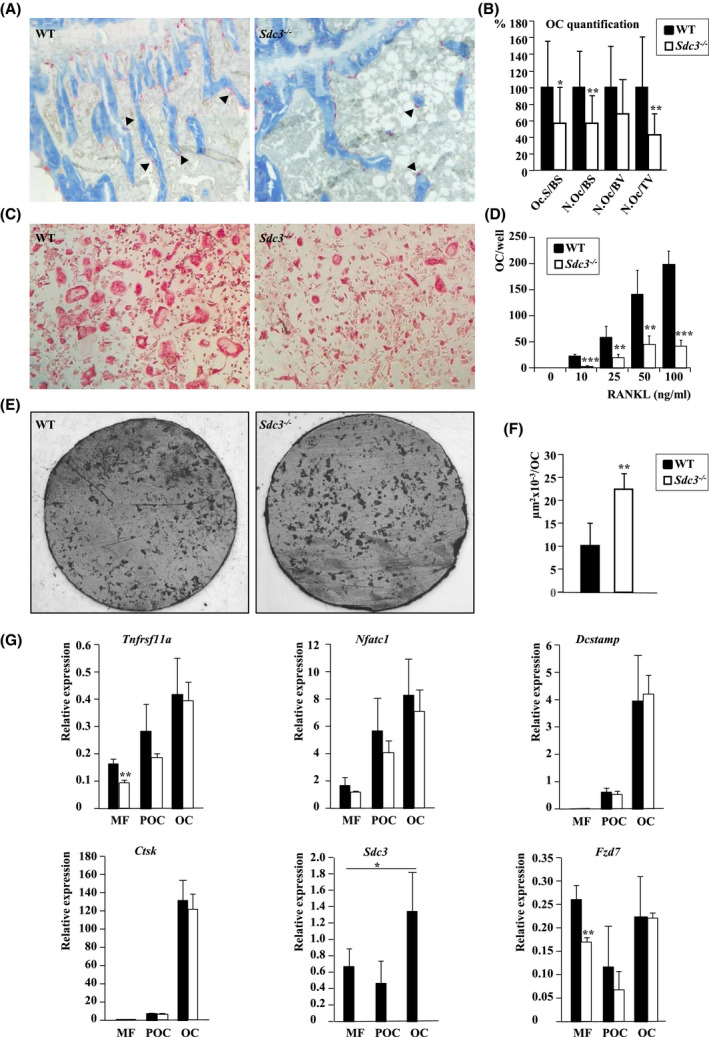
Syndecan‐3 enhances osteoclastogenesis but modulates osteoclastic bone resorption. A, Histology of proximal tibia showing osteoclasts TRAcP‐stained red (indicated by arrowheads), bone counterstained with aniline blue of 3‐month‐old WT (left panel) and *Sdc3^−/−^* (right panel) mice. B, Quantification of TRAcP‐stained osteoclasts on histology of tibiae from 3‐month‐old WT (n = 12) and Sdc3*^−/−^* (n = 12) mice. Oc.S/BS: osteoclast surface/bone surface, N.Oc/BS: number of osteoclasts/bone surface, N.Oc/BV: number of osteoclasts/bone volume, N.Oc/TV: number of osteoclasts/tissue volume. OC: osteoclast. C, Photomicrographs of TRAcP‐stained osteoclasts differentiated in vitro from RANKL‐ and MCSF‐stimulated macrophages of WT (left panel) and *Sdc3^−/−^* (right panel) mice. D, Quantification of osteoclast numbers in RANKL‐ and MCSF‐stimulated macrophage cultures from WT and *Sdc3^−/−^* mice. RANKL stimulation was at 10, 25, 50, and 100 ng/mL. Data shown are from one representative experiment out of five performed (with five replicate wells per mouse cell donor). E, Reflected light microphotographs of dentine slices with areas (in black) resorbed by osteoclasts differentiated from WT (left panel) and *Sdc3^−/−^* (right panel) mice. F, Quantification of resorbed area on dentine slices by osteoclasts from WT and *Sdc3^−/−^* mice (n = 3; four dentine slices per mouse). G, Relative expression of *Tnfrsf11a, Nfatc1, Dcstamp, Ctsk, Sdc3,* and *Fzd7* to *Hmbs* in MCSF‐dependent macrophages (MF), pre‐osteoclasts (POC), and osteoclasts (OC) differentiated from *Sdc3^−/−^* (n = 3) and WT (n = 4) mice. qPCR reactions were performed in triplicate. There was a statistically significant increase in expression of *Tnfrsf11a* (*P < .05*), *Dcstamp* (*P < .01*), *Nfatc1* (*P < .01*), and Ctsk (*P < *.001) between the MF and OC stage in both *Sdc3^−/−^* and WT (not annotated for clarity). Data in B, D, F, and G are shown as mean ± SD, *P < .05, **P < .01, ***P < .001

Bone histomorphometry of 3‐month‐old *Sdc3^−/−^* mice after TRAcP staining for osteoclasts unexpectedly revealed a decrease of 44% in both osteoclast surface and number per bone surface (Oc.S/BS, *P < *.05 and N.Oc/BS, *P < *.01, respectively) vs WT. Likewise, the number of osteoclasts per tissue volume (N.OC/TV) was decreased by 57% in *Sdc3^−/−^* vs WT (*P < *.01; Figure 3A,B, and Table S3).

The rate of osteoclast formation induced by MCSF and RANKL in vitro was significantly lower in osteoclastogenesis cultures derived from *Sdc3^−/−^* vs WT 3‐month‐old mice (Figure 3C,D), confirming the histology findings (Figure 3A,B), and indicating that the decreased osteoclast formation is due to a cell autonomous defect in response to RANKL. Interestingly, cultures of osteoclasts on dentine slices did not show a difference in resorption area between *Sdc3^−/−^* and WT (Figure 3E), however, due to there being fewer *Sdc3^−/−^* osteoclasts vs WT formed per dentine slice (80.6 ± 18.4 vs 141.2 ± 98.5, respectively, *P < *.05), the resorption area per osteoclast was over twofold higher in *Sdc3^−/−^* vs WT cultures, *P < *.01 (Figure 3F). These findings suggest that SDC3 promotes osteoclast differentiation but decreases mature osteoclast resorptive activity.

To gain insight into the mechanistic role of SDC3 in osteoclastogenesis, we examined osteoclast marker gene expression during osteoclast differentiation in vitro. Only at the early, MCSF‐dependent macrophage stage, did we find a significant decrease in the *Tnfrsf11a* encoding RANK in *Sdc3^−/−^* vs WT (Figure 3G), which may explain lower sensitivity of *Sdc3^−/−^* macrophage‐lineage cells to RANKL and consequently impaired osteoclastogenesis. The expression of the major transcription factor inducing osteoclastogenesis, *Nfatc1*, increased during osteoclast differentiation, as did the late osteoclast markers *Dcstamp* and *Ctsk* as expected, with no significant differences between genotypes. Interestingly, *Sdc3* expression significantly increased in osteoclasts vs MCSF‐dependent macrophages, suggesting a role for SDC3 in mature osteoclasts (Figure 3G). Given that during osteoclast formation RANK expression has been shown to be induced by the FZD‐mediated noncanonical WNT signaling pathway ROR/JNK/c‐JUN^32^ and FZD7 is highly expressed,^33^ whereas in *Xenopus* FZD7 associates with SDC4 and RSPO3 in the noncanonical WNT/PCP pathway during endocytosis,^16^ we assessed *Fzd7* expression during osteoclastogenesis. Interestingly, again *Fzd7* expression was significantly lower in *Sdc3^−/−^* MCSF‐dependent macrophages, but not at the pre‐osteoclast or osteoclast stage (Figure 3G). As FZD7 is involved in both canonical and noncanonical WNT‐signaling^16^, ^34^, ^35^ and is likely induced by β‐catenin itself through the TCF/LEF promoter binding site,^36^ taken together our data suggest that SDC3 enhances early stages of osteoclast differentiation via WNT‐signaling and plays a role in mature osteoclasts, however, the exact mechanism of action of SDC3 in osteoclast‐lineage cells is currently unclear.

### 3.4 Low bone volume phenotype in adult *Sdc3^−/−^* mice is due to impaired osteoblast maturation and reduced bone formation

**FIGURE 4:**
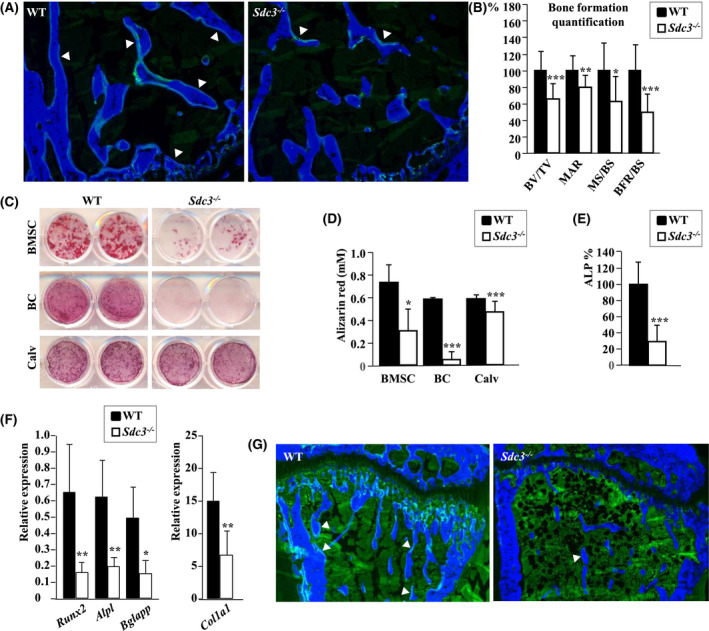
Syndecan‐3 enhances bone formation in adult mice. A, Histomorphometry of tibiae from calcein‐double labeled 3‐month‐old WT (left panel) and *Sdc3^−/−^* (right panel) mice, bone counterstained with calcein blue. Double label is indicated by arrowheads. B, Quantification of bone formation in calcein‐double labeled 3‐month‐old WT (n = 12) and *Sdc3^−/−^* (n = 12) mice. BV/TV: bone volume/tissue volume, MAR: mineral apposition rate, MS/BS: mineralizing surface/bone surface, BFR/BS: bone formation rate/bone surface. C, Photographs of representative WT and *Sdc3^−/−^* osteoblast cultures stained with alizarin red to show mineralization. In top, middle, and bottom row osteoblasts differentiated from bone marrow stromal cells (BMSC), bone chips (BC), and calvariae (Calv), respectively. D, Alizarin red quantification of mineral from cultures of WT and *Sdc3^−/−^* osteoblasts differentiated from BMSC (n = 3, 4 replicate wells each), bone chips (BC, n = 3, 2 replicates each), and calvariae (Calv, n = 3, 2 replicates each) as indicated. E, Alkaline phosphatase (ALP) quantification in WT and *Sdc3^−/−^* osteoblasts grown out from bone chips, data normalized to WT (n = 4, 5 replicates each). F, RNA expression of osteoblast marker genes: *Runx2*, *Alpl* (encoding ALP), *Bglap* (encoding osteocalcin), and *Col1a1* relative to *Hmbs assesse*d by qPCR in osteoblasts grown out of bone chips from WT (n = 3) and *Sdc3^−/−^* (n = 3) mice. G, Histomorphometry of mechanically loaded tibiae from calcein‐double labeled 3‐month‐old WT (left panel) and *Sdc3^−/−^* (right panel) mice, bone counterstained with calcein blue. Double label is indicated by arrowheads. Quantification is shown in Table 1. Data in B, D, E, and F are shown as mean ± SD, *P < .05; **P < .01; ***P < .001

Dynamic histomorphometry of bone formation using calcein‐double label quantification in 3‐month‐old *Sdc3^−/−^* mice revealed a decrease of 35% in BV/TV (*P < *.001), 21% in the mineral apposition rate (MAR, *P < *.01), 36% in the mineralizing surface per bone surface (MS/BS (*P < *.05), and 50% in the bone formation rate per bone surface (BFR/BS (*P < *.001) vs WT (Figure 4A,B, and Table S3) in keeping with low bone formation rate in *Sdc3^−/−^* mice. This was corroborated by a 40% reduction in the serum concentration of the bone formation marker, P1NP, in *Sdc3^−/−^* vs WT mice (Fig. S2). Functional assays confirmed an over two‐ and sixfold reduction in mineralization in *Sdc3^−/−^* vs WT cultures of osteoblasts differentiated from BMSCs and from BCs, respectively (Figure 4C,D). *Sdc3^−/−^* osteoblasts grown out of neonatal calvariae also showed significantly reduced mineralization capacity vs WT (Figure 4C,D). Unsurprisingly, alkaline phosphatase activity was reduced by 70% in *Sdc3^−/−^* vs WT osteoblasts differentiated from BCs in vitro (*P < *.001; Figure 4E), indicating that the low bone formation rate seen in *Sdc3^−/−^* mice on histomorphometry is due to impaired osteoblast function. As the proliferation rate of osteoblasts assessed by the Ki67 assay was higher in *Sdc3^−/−^* compared to WT cultures (9.1 ± 1.7 vs 7.0 ± 1.1, *P < *.05), taken together these data suggest that SDC3 is important for timely maturation of proliferating osteoblasts and normal bone formation and mineralization.

The functional impairment of *Sdc3^−/−^* osteoblasts was corroborated by the finding of downregulated osteoblastic differentiation and activity genes: *Runx2* by 75% (*P <* .01), *Alpl* (encoding alkaline phosphatase) by 70% (*P < *.01), *Bglap* (encoding osteocalcin) by 70% (*P < *.05), and *Col1a1* by 55% (*P < *.01) vs WT (Figure 4F). In addition, we quantified *Sdc1*, *Sdc2,* and *Sdc4* expression in osteoblasts, to assess whether their level might be increased in an effort to compensate for lack of *Sdc3*. This analysis showed expression of all four syndecan genes, with *Sdc4* being the most highly expressed, however, we found no differences in expression of *Sdc1, 2,* and *4* between *Sdc3^−/−^* and WT osteoblasts (Figure S3).

As bone responds with new bone formation to mechanical loading, we assessed this anabolic response in vivo. As expected, in WT mice mechanical loading of the tibia induced an increase in mineralizing surface (MS/BS) and in bone formation rate (BFR/BS) of 41% (*P < *.001) and 48% (*P < *.05), respectively, in the loaded vs non‐loaded tibia. However, in *Sdc3^−/−^* mice the anabolic response to mechanical loading was blunted: the increase in MS/BS and BFR/BS in the loaded vs non‐loaded tibia was only 12% and 21%, respectively (Figure 4G and Table 1), and not statistically significant.

**TABLE 1:** Anabolic response to mechanical loading analyzed by histomorphometry

	WT (n = 7)			*Sdc3^−/−^* (n = 8)		
	Non‐loaded	Loaded	% Change	Non‐loaded	Loaded	% Change
MAR	1.97±0.27	2.04±0.23	4.57±14.66	1.60±0.28	1.65±0.26	3.64±17.10
MS/BS	27.53±8.49	38.37±10.93	40.72±17.83***	16.10±5.22	18.14±7.43	11.65±32.48
BFR/BS	0.54±0.18	0.80±0.30	48.77±29.19*	0.26±0.09	0.31±0.15	20.84±46.76

Note

The right tibia of 10‐week‐old male WT and Sdc3^−/−^ mice was mechanically loaded for 2 weeks as described, whereas the left tibia was not loaded. Anabolic response was quantified by % change in loaded vs non‐loaded tibia and statistical significance calculated using paired Student's t test. Values shown are means ± SD.

Abbreviations: BFR/BS, bone formation rate/bone surface (µm^3^/µm^2^/day); MAR, mineral apposition rate (µm/day); MS/BS, mineralizing surface/bone surface (%).

* *P < *.05;

*** *P < *.001.

### 3.5 Bone marrow adiposity is increased in *Sdc3^−/−^* mice

**FIGURE 5:**
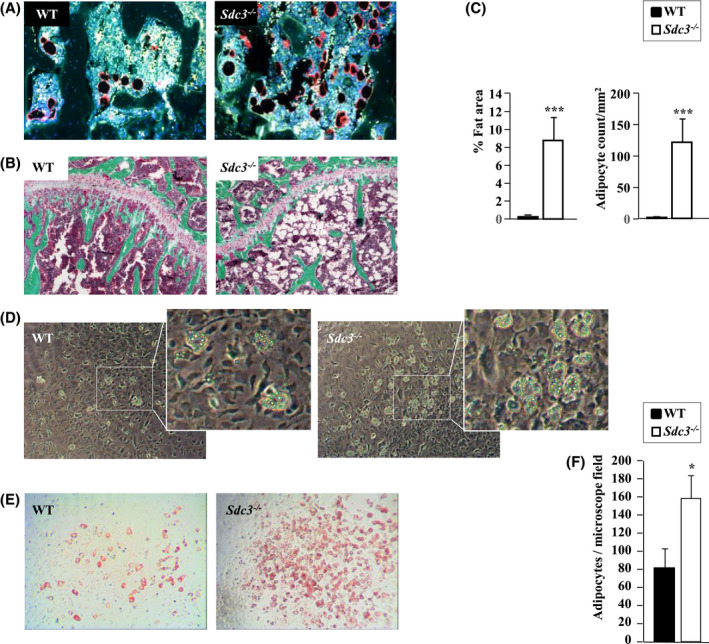
Syndecan‐3 deletion leads to an increase in bone marrow adipose tissue (BMAT). A, Perilipin stain of bone marrow adipocytes (seen as black ovals with a red rim) in proximal tibia from 3‐month‐old WT (left panel) and *Sdc3^−/−^* (right panel) mice. B, Goldner's trichrome stain of proximal tibia from 3‐month‐old WT (left panel) and *Sdc3^−/−^* (right panel) mice, showing oval voids corresponding to adipocytes within the bone marrow. C, Quantification of BMAT area and adipocyte count in WT (n = 6) and *Sdc3^−/−^* (n = 4) mice on histology. D, Representative images of WT (left panel) and *Sdc3^−/−^* (right panel) BMSCs grown in adipo‐osteogenic conditions for 12 days, fixed and visualized by phase‐contrast microscopy at 10x magnification. White rectangles delineate clusters of adipocytes magnified in the smaller panels to the right. E, Representative images of BMSC cultures described in d stained with Oil‐red‐O to visualize adipocytes. Bright field microscopy, magnification 10x. F, Quantification of adipocytes from WT (n = 3) and *Sdc3^−/−^* (n = 3) BMSC cultures described in d and e. Data in c and f are mean ± SD, *P < .05; ***P < .001

As *Sdc3* deletion leads to a bone phenotype mimicking premature osteoporosis, which in humans is typically associated with increased bone marrow adipose tissue (BMAT), we quantified BMAT, seen as empty spaces in the bone marrow on the Goldner's Trichrome stain, after the void areas had been confirmed to contain adipocytes on perilipin staining (Figure 5A). Histomorphometry analysis of tibial and femoral bones from 3‐month‐old mice revealed a 60‐fold increase in bone marrow adipocyte number and area in *Sdc3^−/−^* compared to WT (*P < *.001, Figure 5B,C). In vitro, a twofold increase in adipocyte formation was observed in *Sdc3^−/−^* vs WT BMSCs cultured in adipo‐osteoblastogenesis conditions (Figure 5D‐F) and adipocytes formed 1‐2 days earlier in *Sdc3^−/−^* than in WT cultures. Thus, deletion of *Sdc3* leads to a premature osteoporosis‐like phenotype characterized by not only low bone volume and fragility, but also increased BMAT, possibly due to a preferential switch from osteoblastogenesis to adipogenesis at a progenitor level.

### 3.6 Syndecan‐3 enhances the canonical WNT signaling pathway in osteoblasts

Given that a common signaling pathway enhancing osteoblastogenesis, mediating anabolic response to mechanical loading and in cartilage critical for normal growth plate development is WNT mediated,^4^, ^37^ we hypothesized that SDC3 enhances WNT signaling.

**FIGURE 6:**
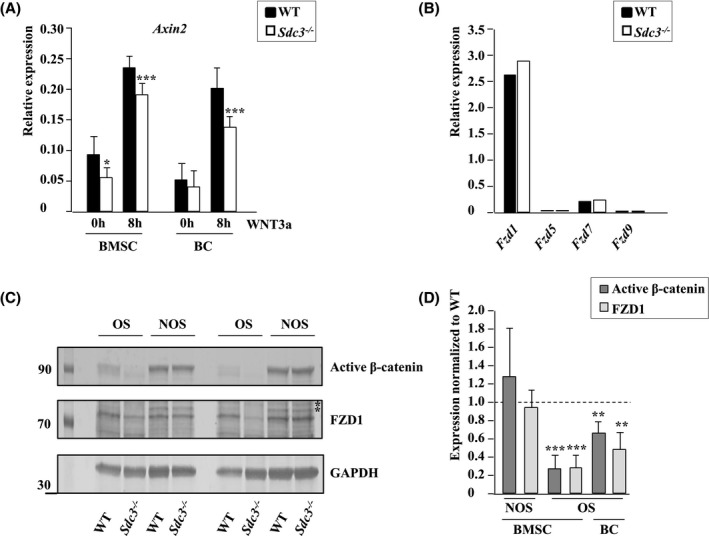
Syndecan‐3 deletion enhances Frizzled 1 degradation and impairs canonical WNT signaling in osteoblasts. A, Osteoblasts were differentiated from bone marrow mesenchymal stromal cells (BMSC) or bone chips (BC) from *Sdc3^−/−^* (n = 3) or WT (n = 3) mice and grown in osteogenic conditions. Sixteen hours prior to stimulation with WNT3a FCS was replaced with TCM and *Axin2* expression relative to *Hmbs* was quantified by qPCR in triplicate. Values shown are means ± SD. *P < .05; **P < .01; ***P < .001. B, Osteoblasts were differentiated from BC and grown until confluence. *Fzd1, 5, 7, and 9* expression relative to *Hmbs* was quantified by qPCR in triplicate (n = 1). C, Osteoblasts were differentiated from *Sdc3^−/−^* (n = 5) and WT (n = 5) BMSCs and grown in non‐osteogenic (NOS) or osteogenic (OS) conditions. Cell lysates were immunoblotted for non‐phospho (active) β‐catenin, FZD1, and GAPDH. Western blot shows two representative experiments out of five. Asterisks indicate nonspecific bands. D, Immunoblot quantification of non‐phospho (active) β‐catenin and FZD1 protein expression in *Sdc3^−/−^* osteoblasts differentiated from *Sdc3^−/−^* (n = 5) and WT (n = 5) BMSCs (as per c) or BCs (*Sdc3^−/−^* n = 5, WT n = 5) grown in NOS or OS conditions until confluent. All β‐catenin and FZD1 bands were normalized to GAPDH and *Sdc3^−/−^* data were normalized to respective WT. Data are shown as means ± SD. **P < .01; ***P < .001

To assess the effect of *Sdc3* deletion on canonical WNT‐signaling, osteoblasts were stimulated with WNT3a, which is known to enhance osteoblastogenesis,^38^, ^39^ and the expression of the main β‐catenin target gene *Axin2* was quantified by qPCR. In *Sdc3^−/−^* osteoblast cultures there was a significant reduction in *Axin2* expression compared to WT controls regardless of whether the osteoblasts were grown out of BCs or differentiated from BMSCs, which was mostly apparent after stimulation with WNT3a (Figure 6A) and in keeping with decreased canonical WNT‐signaling in the absence of SDC3.

Thus, we further assessed the main canonical WNT‐signaling pathway components at mRNA level and found no difference in expression of *Lrp5* or *6*, *Lgr4‐6*, *Znrf3,* and *Rnf43*, in *Sdc3^−/−^* vs WT osteoblasts (data not shown). From the *Fzds* involved in canonical WNT signaling (ie, *Fzd1*, *5*, *7,* and *9*), *Fzd1* expression in osteoblasts was highest (Figure 6B) in keeping with previous reports.^40^ As FZD1 enhances osteoblast‐mediated mineralization,^41^ we quantified FZD1 and non‐phospho (active) β‐catenin at protein level and found both reduced by 72% and 73%, respectively, in *Sdc3^−/−^* vs WT (*P < *.001) in BMSC‐derived osteoblasts grown in osteogenic conditions (Figure 6C,D). In osteoblasts derived from bone‐chips, FZD1 and active β‐catenin were reduced by 52% (*P < *.01) and 34% (*P < *.01), respectively, in *Sdc3^−/−^* vs WT (Figure 6C,D). In contrast, active β‐catenin levels were considerably higher in BMSCs cultured in non‐osteogenic conditions, and there was no difference in either FZD1 or active β‐catenin levels between *Sdc3^−/−^* and WT in these cells. Taken together, these data suggest that in osteoblasts SDC3 enhances the canonical WNT‐signaling pathway by stabilizing FZD1.

### 3.7 Osteoblast‐specific expression of Syndecan‐3 rescues *Sdc3^−/−^* low bone volume phenotype

**FIGURE 7:**
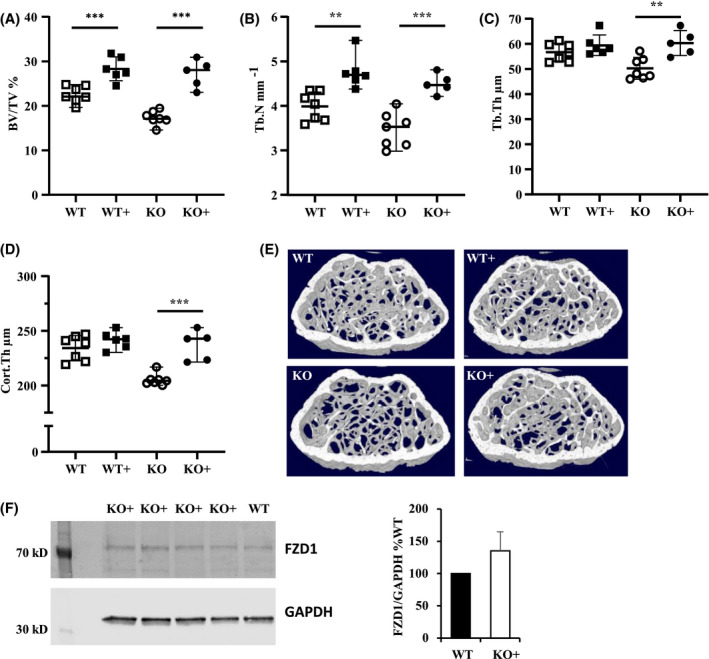
Osteoblast‐specific overexpression of Syndecan‐3 increases bone volume. A‐D, µCT analysis of distal femurs of 3‐month‐old male mice. WT: wild type (N = 7), KO: *Sdc3^−/−^* (N = 7), WT+: WT with osteoblast‐specific overexpression of *Sdc3* driven by the 2.3kb *Col1a1* promoter (N = 6), KO+: *Sdc3^−/−^* with osteoblast‐specific overexpression of *Sdc3* (N = 5). BV/TV: bone volume, Tb.Th: trabecular thickness, Tb.N: trabecular number, Cort.Th: cortical thickness. **P < .01; ***P < .001. E, Cross‐sectional image of a 3D µCT reconstruction of distal femurs of WT, KO, WT+, and KO+ mice representing the volumes used for analysis of trabecular bone parameters. F, Immunoblot (left panel) and quantification (right panel) of the effect of *Sdc3* driven by the 2.3kb *Col1a1* promoter in *Sdc3^−/−^* (KO+) on FZD1 protein level in osteoblasts grown out of bone chips. KO+: n = 4 and WT: n = 1. FZD1 bands were normalized to GAPDH and KO + data were normalized to WT. Data are shown as means ± SD

Given the indicative enhancing role of SDC3 on osteoblast function we generated a transgenic mouse expressing *Sdc3* driven by the 2.3 kb *Col1a1* promoter on *Sdc3* null background (*Col1a1‐Sdc3,* KO+). At 3 months of age the trabecular low bone volume phenotype of *Sdc3^−/−^* mice was fully reversed by osteoblast‐specific overexpression of *Sdc3*, with *Col1a1‐Sdc3* mice displaying a 50% higher BV/TV (*P < *.001*)* than *Sdc3^−/−^* (Figure 7 and Table S4a). Cortical thickness was 15% (*P < *.001) higher in the *Col1a1‐Sdc3* vs *Sdc3^−/−^* mice as well, and the increase in cortical thickness was due to an increase in periosteal perimeter (*P <* .05), and leading to a 24% (*P <* .05) increase in polar moment of inertia (Figure 7 and Table S4B). Thus, Sdc3 expressed in osteoblasts plays an essential role in trabecular and cortical bone homeostasis. Interestingly, overexpression of *Col1a1‐Sdc3* in WT mice (WT+) led to an approximately 25% increase in BV/TV (*P < *.001) vs WT (Figure 7. and Table S4A), although the 3% increase in cortical thickness in WT+ mice was not statistically significant. Finally, when we analyzed the protein levels of FZD1 in osteoblasts by western blot, we found that overexpression of *Sdc3* in *Sdc3* null osteoblasts increased FZD1 to levels even higher than in wild‐type osteoblasts (Figure 7F). Our finding that osteoblast lineage‐specific overexpression increases bone volume in WT mice further demonstrates the anabolic role of Sdc3 in bone.

## 4 DISCUSSION

Here, we have identified a novel anabolic role for SDC3 in bone. Deletion of *Sdc3* leads to low bone volume, increased bone fragility, and increased bone marrow fat in young adult mice, a premature osteoporosis‐like phenotype. This phenotype is mediated primarily by the lack of SDC3 in osteoblasts, as the low bone volume phenotype of *Sdc3^−/−^* is rescued by *Col1a1*‐driven *Sdc3* expression.

Interestingly, SDC3 differentially affects pre‐ and postnatal long bone growth. *Sdc3* deletion resulted in increased long bone elongation in utero, in keeping with the known restrictive role of SDC3 at two stages of skeletal development. First, during the condensation stage of early pre‐cartilaginous skeletogenesis SDC3 restricts the size of mesenchymal cell aggregates and promotes the differentiation step.^18^, ^42^, ^43^ Second, during limb elongation SDC3 is thought to set boundaries at the metaphyseal and epiphyseal perichondrium and periosteum.^42^, ^44^ Thus, deletion of *Sdc3* would allow for a relative expansion of the cartilaginous skeleton, explaining the longer bones seen early postnatally in *Sdc3^−/−^* mice. Moreover, it has been previously shown that SDC3 regulates proliferation and maturation of chondrocytes in the growth plate during embryonic long bone development^20^, ^45^, ^46^ and restricts chondrogenic differentiation in vitro.^31^ Our data indicate that SDC3's regulatory role in chondrocytes extends into adulthood as *Sdc3* deletion leads at 3 months of age to a thin growth plate with disorganized columns of chondrocytes within the proliferating zone, which is associated with mild, but significant long bone growth stunting compared to the reverse seen in utero. This growth plate abnormality may be due to defective chondrogenesis at stem cell level in the growth plate resting zone,^47^, ^48^ and/or at the proliferation and differentiation stages of chondrocytes,^19^, ^20^ in which extracellular matrix, with which transmembrane HSPGs interact, is thought to play a role. Further investigations are in progress to assess the exact role of SDC3 in the regulation of chondrocytes within the growth plate and in longitudinal bone growth.

Significantly, the relatively large total long bone volume of *Sdc3^−/−^* pups is not only not maintained into adulthood but is lower than that of WT mice already at 2 weeks of age. Furthermore, the trabecular bone volume (BV/TV) becomes significantly lower in *Sdc3* *^−/−^* than WT mice at 2 months and remains low. While the shortening of the long bones in *Sdc3^‐/‐^* adult mice reflects a thinner and morphologically abnormal growth plate, the low bone volume phenotype is unexpected. However, a similar long bone phenotype has been reported in young adult mice deficient in the heparin‐binding growth molecule (HB‐GAM, also known as osteoblast stimulating factor 1 [OSF1] and pleiotrophin), expressed at the growth plate and around osteocytes in bone.^49^, ^50^ Thus, our findings indicate that SDC3, like HB‐GAM, mediates an osteogenic effect in response to mechanical loading, given that SDC3 likely serves as receptor for HB‐GAM on osteoblasts, as has been previously shown in neurons.^51^

Although osteoclastogenesis is decreased in *Sdc3^−/−^* mice (like in *HB‐GAM^−/−^*), the relative scarcity of osteoclasts is likely offset by increased osteoclast‐mediated bone resorption, which may be contributory to the low bone volume phenotype. The latter finding contrasts with the lack of effect of HB‐GAM on osteoclastic bone resorption.^49^ Our observation of a gradual increase in SDC3 expression during normal osteoclast differentiation suggests its importance for osteoclast maturation and/or function. Indeed, reduced expression of *Tnfrsf11a* encoding RANK and *Fzd7* in MCSF‐dependent *Sdc3^−/−^* macrophages, is reflected by reduced sensitivity to RANKL and suggests that SDC3 may enhance pathways targeting *Fzd7*. These include upstream from *Fzd7* the canonical β‐catenin mediated WNT^36^, ^52^ and the noncanonical WNT signaling mediated by FZD via RAC/JNK/c‐Jun inducing expression of RANK.^32^ On the other hand, it is tempting to speculate that SDC3 may associate with FZD7 during cell polarization, not unlike SDC4 during the planar cell polarity (PCP) process in Xenopus, which also leads to activation of JNK.^16^ Furthermore, in human breast epithelial cells FZD7 is induced by NOTCH signaling,^53^ which is known to cross talk with SDC3 during myogenesis,^54^ thus, further research is currently ongoing exploring the SDC3‐NOTCH cross talk in bone. Our findings are not explained by the lack of the SDC3 ectodomain in the light of Kim's and colleagues recent report of a suppressive effect of SDC1‐4 ectodomains on osteoclastogenesis and of SDC1, 2, and 4 (but not SDC3) ectodomains on osteoclast‐mediated bone resorption.^55^

The *Sdc3^−/−^* mouse low bone volume phenotype primarily reflects impaired osteoblastic bone formation, which is associated with increased bone marrow adiposity, suggesting either a preferential switch at the mesenchymal progenitor level from osteoblastogenesis to adipogenesis, or potential de‐differentiation of osteoblasts toward the adipocyte lineage. It is unlikely that systemic factors play a significant role in this aspect of the phenotype, as at a cellular level there is increased adipocyte formation seen in *Sdc3^−/−^* BMSCs vs WT in vitro. Indeed, the delay in *Sdc3^−/−^* osteoblast differentiation suggests a degree of immaturity of differentiated *Sdc3^−/−^* osteoblasts, which display impaired ability to not only form, but also fully mineralize newly formed bone. The blunted anabolic response of bone to mechanical loading in *Sdc3^−/−^* mice is not due to muscle dysfunction as deletion of *Sdc3* in fact improves muscle homeostasis, regeneration, and aging, in both healthy and dystrophic mice.^56^ Moreover, *Sdc3^−/−^* mice are no different from WT in regard to endurance training performance.^56^ Finally, osteoblast‐specific overexpression of *Sdc3* both in the *Sdc3^−/−^* and WT mice significantly increases bone volume, trabecular number (and cortical thickness in *Sdc3^−/−^*), which demonstrates that the bone anabolic effect of SDC3 is osteoblast‐dependent. However, the critical step affected by SDC3 in the process of osteoblastogenesis remains to be elucidated.

As the key pathways regulating skeletal development, inducing osteoblastogenesis,^4^ inhibiting adipogenesis,^57^, ^58^ and in cartilage inducing chondrocyte hypertrophy and regulating the morphology of the growth plate^37^, ^59^ involve WNTs,^4^ we focused our investigations on the canonical WNT signaling. To our knowledge, involvement of SDC3 in WNT signaling has not been reported previously, although SDC1, 2, and 4 are co‐receptors in WNT signaling.^17^ Given the severity of the *Sdc3^−/−^* mouse bone phenotype, clearly SDC1, 2, and 4 are unable to compensate for lack of SDC3, indicating its specific role in the acquisition of bone volume and maintenance of normal bone homeostasis in adult mice, corroborated by the rescue of the *Sdc3^−/−^* low bone volume phenotype by *Col1a1*‐driven *Sdc3*.

We have consistently found decreased expression of *Axin2* in *Sdc3^−/−^* osteoblasts indicating impaired β‐catenin signaling. Out of the *Fzds* classically involved in canonical WNT signaling, that is, *Fzd1*, *5*, *7,* and *9*,^17^ we found *Fzd1* expression in osteoblasts to be highest, as reported previosuly.^40^ Given that FZD1 not only mediates osteoblast differentiation, but also bone mineralization^41^ and that *Sdc3^−/−^* osteoblasts show impairment in both these functions, our findings of decreased both FZD1 and active β‐catenin in *Sdc3^−/−^* osteoblasts may explain the *Sdc3^−/−^* phenotype. Furthermore, as *Fzd1* RNA expression is not affected in *Sdc3^−/−^* osteoblasts, whereas at the protein level FZD1 is significantly decreased in *Sdc3^−/−^* osteoblasts, the most plausible explanation is that in the absence of SDC3, FZD1 undergoes rapid degradation, leading to decreased β‐catenin signaling. Indeed, overexpression of the *Col1a1*‐driven *Sdc3* restores the FZD1 levels. Thus, we propose that in osteoblasts SDC3 enhances canonical WNT signaling and likely serves as co‐receptor which stabilizes FZD1, however, the exact molecular mechanism of this interaction remains to be explored. Interestingly, active β‐catenin levels were considerably higher in BMSCs grown under non‐osteogenic conditions than in those cultured for 7 days in osteogenic conditions, findings similar to those previously reported in a human pre‐osteoblast SV‐HFO cell line.^40^ This may indicate that during osteoblast differentiation active β‐catenin levels, already low, are more critical and tightly controlled. Thus, any changes in FZD1 levels may have a relatively more pronounced effect on β‐catenin signaling.

Although our findings indicate that SDC3 enhances canonical WNT signaling in the osteoblast lineage and both canonical and noncanonical WNT signaling in osteoclast lineage, other pathways remain to be investigated. These, in addition to the remaining WNT signaling pathways, include FGF, BMP, and NOTCH, cross talk of which orchestrates osteoblast differentiation,^60^ given that SDCs have been shown to partake in all these pathways.^61^ Importantly, interaction of SDC3 with HB‐GAM has been shown to regulate osteoblast recruitment to sites of increased bone formation^50^ and osteocytic HB‐GAM expression is upregulated after mechanical loading,^49^ thus, investigation of this pathway in the *Sdc3^−/−^* mouse is ongoing.

In summary, SDC3 differentially affects pre‐ and postnatal bone development. SDC3 deletion leads to a low bone volume phenotype associated with high BMAT in young adult mice. The anabolic effect of SDC3 on bone is mediated through canonical WNT signaling in osteoblasts, likely through stabilization of FZD1. *Col1a1*‐driven *Sdc3* overexpression not only rescues the low bone volume phenotype in *Sdc3^−/−^*, but also increases bone volume in WT mice. Thus, SDC3 may be a novel therapeutic target for anabolic drug development for prevention or treatment of osteoporosis.

## CONFLICT OF INTEREST

The authors declare no conflict of interests.

## AUTHOR CONTRIBUTIONS

A. Daroszewska and RJ van 't Hof designed the experiments with input from A. Butcher, A. Pisconti,^3^, ^4^ G. Bou‐Gharios, and B. Poulet; FM Johnson de Sousa Brito, A. Butcher, A. Pisconti,^1^ G. Charlesworth, C. Sperinck, K. Liu, and RJ van 't Hof performed the experiments and acquired the data; FM Johnson de Sousa Brito, AB, A. Pisconti,^3^, ^4^ B. Poulet, MS di Mase, G. Bou‐Gharios, RJ van 't Hof, and A. Daroszewska interpreted and analyzed the data; A. Daroszewska and RJ van 't Hof wrote the manuscript, which was critically revised by all coauthors.

## Supporting information

Supplementary Material
