# Supplementary material for: Syndecan‐3 enhances anabolic bone formation through WNT signaling
Source: FASEB J. 2021 Mar 26;35(4):e21246. doi: 10.1096/fj.202002024R (PMC8251628; doi:10.1096/fj.202002024R)
Supplement: Supplementary file 1 — Supplementary Material [file FSB2-35-0-s001.docx]

**Supplementary Fig. 1**

**Creation of a transgenic mouse with osteoblast-specific overexpression of Sdc3.**

**(a)** Targeting vector used for pro-nuclear injection. 2.3kb Col1a1: the osteoblast specific *Col1a1* promoter; Sdc3: cDNA of *Sdc3* mRNA; cMyc: cMyc tag; mCherry: mCherry coding sequence; pA: polyadenylation sequence from the bovine growth hormone gene; neoR: neomycin resistance gene. For1 and Rev1: Forward and reverse primers amplifying a 212bp fragment from the *Col1a1* promoter to the Sdc3 coding sequence. For2 and Rev2: Forward and reverse primers amplifying a 287bp fragment from the Sdc3 coding sequence to the mCherry coding sequence.

**(b)** Genotyping results for three transgenic mice using primer sets 1 and 2. The positive control was the plasmid containing the targeting vector. Arrows indicate bands of the expected size. Because of a cleaner PCR without any non-specific bands, primer set 2 (Sdc3 to mCherry) was used for routine genotyping.

**Supplementary Fig. 2**

*

**P1NP serum concentration in WT and Sdc3^-/-^ mice**

Serum was obtained from 3-month-old male WT (n=5) and *Sdc3^-/-^* (n=5) mice and P1NP concentration quantified. Briefly, whole blood was obtained by cardiac puncture at time of sacrifice and serum was stored at -80°C. Serum concentration of the bone formation marker P1NP was determined in one batch using the Rat/mouse P1NP EIA from Ids (UK) and following manufacturer’s instructions. Horizontal bar indicates mean, * *p<0.05*.

**Supplementary Fig. 3**

**
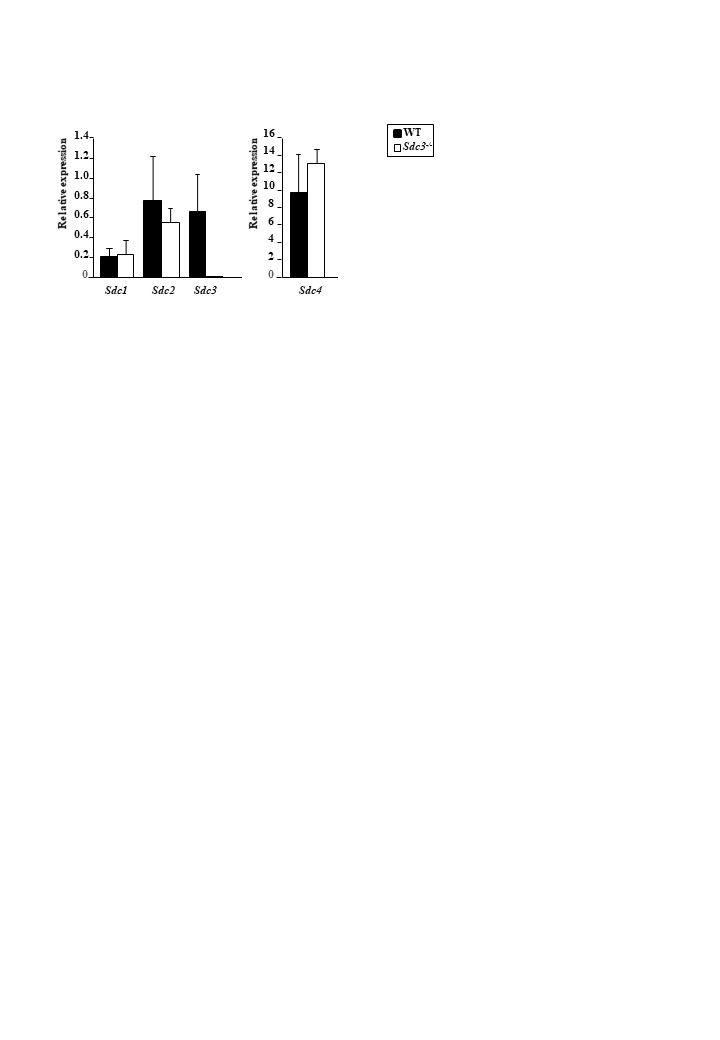
**

**Syndecan expression in osteoblasts**

RNA expression of *Sdc1*, *Sdc2*, *Sdc3* and *Sdc4* relative to *Hmbs assesse*d by qPCR in osteoblasts grown out of bone chips from WT (n=3) and *Sdc3^-/-^* (n=3) mice. Data are shown as mean±SD.

**Supplementary Table 1**

Primer probe sets

| **Roche Universal Probe Library primer probe sets** | | |  |
| --- | --- | --- | --- |
| *Gene* | *Forward (5’-3’)* | *Reverse (5’-3’)* | *Probe ID* |
| *Hmbs* | TCCCTGAAGGATGTGCCTAC | AAGGGTTTTCCCGTTTGC | 79 |
| *Tnfrsf11a* | GTGCTGCTCGTTCCACTG | AGATGCTCATAATGCCTCTCCT | 25 |
| *Dcstamp* | ACAAACAGTTCCAAAGCTTGC | GACTCCTTGGGTTCCTTGCT | 11 |
| *Nfatc1* | TCCAAAGTCATTTTCGTGGA | TTTGCTTCCATCTCCCAGAC | 50 |
| *Ctsk* | CGAAAAGAGCCTAGCGAACA | TGGGTAGCAGCAGAAACTTG | 18 |
| *Sdc3* | CAGCTCCCTCAGAAGAGCATA | CAGGAAGGCAGCGAAGAG | 12 |
| *Runx2* | CCACAAGGACAGAGTCAGATTACA | TGGCTCAGATAGGAGGGGTA | 60 |
| *Alpl* | AAGGCTTCTTCTTGCTGGTG | GCCTTACCCTCATGATGTCC | 16 |
| *Bglap* | AGACTCCGGCGCTACCTT | CTCGTCACAAGCAGGGTTAAG | 32 |
| *Col1a1* | CTCCTGGCAAGAATGGAGAT | AATCCACGAGCACCCTGA | 79 |
|  | |  |  |
| **Roche Realtime Ready Primer Probe Sets** | |  |  |
| *Gene* | *Primer Probe Set ID* |  |  |
| *Fzd1* | 317881 |  |  |
| *Fzd5* | 300412 |  |  |
| *Fzd9* | 317863 |  |  |
| *Fzd7* | 310548 |  |  |
| *Znrf3* | 318785 |  |  |
| *Axin2* | 315714 |  |  |
| *Ctnnb1* | 300053 |  |  |
| *Lgr4* | 318678 |  |  |
| *Lgr5* | 310756 |  |  |
| *Lgr6* | 318676 |  |  |
| *Lrp5* | 310507 |  |  |
| *Lrp6* | 310543 |  |  |
|  | |  |  |
| **Thermo-Fisher Taqman Primer Probe Sets** | |  |  |
| *Gene* | *Primer Probe Set ID* |  |  |
| *Rnf43* | Mm00552558_m1 |  |  |
| *Sdc1* | Mm00448918_m1 |  |  |
| *Sdc2* | Mm04207492_m1 |  |  |
| *Sdc4* | Mm00488527_m1 |  |  |

**Supplementary Table 2a**

Analysis of trabecular bone using µCT.

| **Males** | | | | | | |
| --- | --- | --- | --- | --- | --- | --- |
| Proximal Tibia | BV/TV  % | Tb.Th  µm | Tb.Sp  µm | Tb.N  1/mm | Tb.Pf  1/mm | SMI |
| WT (N=12) | 19.67±2.34 | 55.94±3.36 | 170.35±19.46 | 3.35±0.49 | 16.89±2.12 | 1.74±0.17 |
| Sdc3^-/-^ (N=13) | 13.08±1.97^***^ | 48.88±1.92^***^ | 186.79±10.71^*^ | 2.67±0.34^***^ | 25.31±3.15^***^ | 2.08±0.14^***^ |
| Distal Femur | | | | | | |
| WT (N=12) | 20.09±2.66 | 57.37±4.22 | 181.19±19.06 | 3.52±0.52 | 17.93±2.59 | 1.62±0.22 |
| Sdc3^-/-^ (N=11) | 13.58±2.84^***^ | 49.53±5.02^***^ | 198.46±14.13^*^ | 2.73±0.42^**^ | 22.94±4.13^***^ | 1.93±0.19^**^ |
| **Females** | | | | | | |
| Proximal Tibia | BV/TV  % | Tb.Th  µm | Tb.Sp  µm | Tb.N  1/mm | Tb.Pf  1/mm | SMI |
| WT (N=8) | 13.33±1.21 | 56.05±3.70 | 258.22±24.96 | 2.38±0.22 | 24.63±1.85 | 2.13±0.11 |
| Sdc3^-/-^ (N=7) | 6.02±2.50^***^ | 58.35±4.56 | 372.17±90.19^**^ | 1.05±0.46^***^ | 29.90±8.06 | 2.53±0.43^*^ |
| Distal Femur | | | | | | |
| WT (N=8) | 9.46±0.85 | 58.01±1.83 | 295.84±25.03 | 1.63±0.36 | 26.47±1.46 | 2.39±0.09 |
| Sdc3^-/-^ (N=7) | 5.30±1.76^***^ | 49.83±4.22^***^ | 367.29±79.87^*^ | 1.07±0.15^**^ | 32.47±3.52^***^ | 2.43±0.16 |

Morphometry of proximal tibiae and distal femur of 3-month old male and female *Sdc3^-/-^* and WT mice analysed using µCT. BV/TV: bone volume, Tb.Th: trabecular thickness, Tb.Sp: trabecular separation, Tb.N: trabecular number, Tb.Pf: trabecular pattern factor, SMI: structure model index. Values shown are means±SD, **p<0.05,* **p<0.01* and ****p<0.001*

**Supplementary Table 2b**

Analysis of cortical bone using µCT.

| Tibia | Cort.Th  µm | E.Pm  mm | P.Pm  mm | Polar MMI  mm^4^ |
| --- | --- | --- | --- | --- |
| WT (N=12) | 259.82±15.56 | 2.34±0.15 | 4.24±0.52 | 0.20±0.07 |
| Sdc3^-/-^ (N=10) | 237.30±9.7^**^ | 2.23±0.08 | 3.86±0.10^*^ | 0.13±0.05^*^ |
| Femur | | | | |
| WT (N=9) | 224.80±14.53 | 4.76±0.32 | 5.89±0.21 | 0.56±0.10 |
| Sdc3^-/-^ (N=11) | 201.15±13.64^***^ | 4.21±0.23^***^ | 5.54±0.11^**^ | 0.44±0.07^**^ |

Morphometry of tibial and femoral cortex of 3-month old male *Sdc3^-/-^* and WT mice analysed using µCT. C.Th: cortical thickness, E.Pm: endosteal perimeter, P.Pm: periosteal perimeter, MMI: mean moment of inertia. Values shown are means±SD, **p<0.05,* **p<0.01* and ****p<0.001*

**Supplementary Table 3**

Dynamic bone histomorphometry of *Sdc3^-/-^* mice

|  | WT (n=12) | *Sdc3^-/-^* (n=12) |
| --- | --- | --- |
| BV/TV (%) | 17.13±3.95 | 11.19±3.42^***^ |
| MAR (µm/day) | 1.93±0.36 | 1.53±0.27^**^ |
| MS/BS (%) | 31.09±11.05 | 19.60±9.47^*^ |
| BFR/BS (µm^3^/µm^2^/day) | 0.60±0.20 | 0.30±0.14^***^ |
| Oc.S/BS (%) | 10.52±5.74 | 5.86±4.57^*^ |
| N.Oc/BS (mm^-1^) | 6.50±2.75 | 3.62±2.12^**^ |
| N.Oc/TV (mm^-2^) | 51.71±30.80 | 22.15±12.01^**^ |

Tibias from 3-month-old male WT and *Sdc3^-/-^* mice (n=12) were analysed using histomorphometry. Values shown are means ± SD. Significance (*Sdc3^-/-^* vs WT mice) is denoted by **p<0.05*, ***p<0.01* and ****p<0.001.* BV/TV: bone volume per tissue volume; MAR: mineral apposition rate; MS/BS: mineralising surface per bone surface; BFR/BS: bone formation rate per bone surface; Oc.S/BS: osteoclast surface per bone surface; N.Oc/BS: number of osteoclasts per bone surface; N.Oc/TV: number of osteoclasts per tissue volume.

**Supplementary Table 4a**

Analysis of trabecular bone of KO+ and WT+ using µCT.

| Distal Femur | BV/TV  % | Tb.Th  µm | Tb.Sp  µm | Tb.N  1/mm | Tb.Pf  1/mm | SMI |
| --- | --- | --- | --- | --- | --- | --- |
| KO (N=7) | 17.32±1.58 | 50.23±4.36 | 169.90±11.76 | 3.46±0.38 | 20.2±1.7 | 1.81±0.12 |
| KO+ (N=5) | 27.20±3.15*** | 60.33±4.99** | 145.11±5.85** | 4.50±0.22*** | 15.34±1.62*** | 1.51±0.06*** |
| WT (N=7) | 22.56±1.88 | 56.65±3.53 | 160.40±11.06 | 3.99±0.32 | 17.03±2.67 | 1.59±0.17 |
| WT+ (N=6) | 28.32±2.68*** | 59.43±4.15 | 142.00±7.69** | 4.77±0.37** | 14.33±2.05 | 1.41±0.15 |

Morphometry of distal femur of 3-month old male mice: *Sdc3^-/-^*(KO), *Col1a1-Sdc3* on Sdc3 null background (KO+), WT and *Col1a1-Sdc3* on WT background (WT+), analysed using µCT. BV/TV: bone volume, Tb.Th: trabecular thickness, Tb.Sp: trabecular separation, Tb.N: trabecular number, Tb.Pf: trabecular pattern factor, SMI: structure model index. Values shown are means±SD, **p<0.05,* ***p<0.01* and ****p<0.001versus Col1a2-Sdc3 negative.*

**Supplementary Table 4b**

Analysis of cortical bone of KO+ and WT+ using µCT.

| Femur | Cort.Th  µm | E.Pm  mm | P.Pm  mm | Polar MMI  mm^4^ |
| --- | --- | --- | --- | --- |
| KO (N=7) | 205.19±5.46 | 4.06±0.08 | 5.36±0.10 | 0.44±0.04 |
| KO+ (N=5) | 236.80±13.80*** | 4.11±0.15 | 5.60±0.21* | 0.54±0.08* |
| WT (N=7) | 234.13±11.41 | 4.11±0.20 | 5.56±0.20 | 0.53±0.07 |
| WT+ (N=6) | 241.46±7.91 | 4.26±0.17 | 5.75±0.16 | 0.60±0.06 |

Morphometry of femoral cortex of 3-month old male mice: *Sdc3^-/-^*(KO), *Col1a1-Sdc3* on Sdc3 null background (KO+), WT and *Col1a1-Sdc3* on WT background (WT+), analysed using µCT. C.Th: cortical thickness, E.Pm: endosteal perimeter, P.Pm: periosteal perimeter, MMI: mean moment of inertia. Values shown are means±SD, **p<0.05* and ****p<0.001*
